# Tick-Borne Encephalitis Virus Habitats in North East Germany: Reemergence of TBEV in Ticks after 15 Years of Inactivity

**DOI:** 10.1155/2014/308371

**Published:** 2014-07-08

**Authors:** Silvius Frimmel, Anja Krienke, Diana Riebold, Micha Loebermann, Martina Littmann, Karin Fiedler, Christine Klaus, Jochen Süss, Emil Christian Reisinger

**Affiliations:** ^1^Department of Tropical Medicine, Infectious Diseases and Nephrology, University of Rostock Medical School, Ernst-Heydemann-Straße 6, 18057 Rostock, Germany; ^2^Health Department of the State of Mecklenburg-West Pomerania, 18055 Rostock, Germany; ^3^Friedrich-Loeffler-Institute Jena, National Reference Laboratory for Tick-Borne Diseases, 07743 Jena, Germany; ^4^Tick Information Center, 07646 Lippersdorf, Germany

## Abstract

The incidence of tick-borne encephalitis has risen in Europe since 1990 and the tick-borne encephalitis virus (TBEV) has been documented to be spreading into regions where it was not previously endemic. In Mecklenburg-West Pomerania, a federal state in Northern Germany, TBEV was not detectable in over 16,000 collected ticks between 1992 and 2004. Until 2004, the last human case of TBE in the region was reported in 1985. Following the occurrence of three autochthonous human cases of TBE after 2004, however, we collected ticks from the areas in which the infections were contracted. To increase the chance of detecting TBEV-RNA, some of the ticks were fed on mice. Using nested RT-PCR, we were able to confirm the presence of TBEV in ticks for the first time after 15 years. A phylogenetic analysis revealed a close relationship between the sequences we obtained and a TBEV sequence from Mecklenburg-East Pomerania published in 1992 and pointed to the reemergence of a natural focus of TBEV after years of low activity. Our results imply that natural foci of TBEV may either persist at low levels of activity for years or reemerge through the agency of migrating birds.

## 1. Introduction 

Tick-borne encephalitis (TBE) is the most widespread arthropod-borne viral disease in central Europe. The TBE virus belongs to the genus* Flavivirus* (Fam.* Flaviviridae*), which has three different subtypes: the European subtype, transmitted by* Ixodes ricinus*, and the Siberian subtype and the Far Eastern subtype, both transmitted by* Ixodes persulcatus*. The European subtype is found in all European countries except the Benelux and Great Britain [1, 2].

The incidence of TBE in Europe has risen dramatically since 1990 [3–5]. Comparing the periods from 1974 to 1983 and 1994 to 2003, the average increase in TBE infections in humans in ten European countries was 311% [5]. In Germany, the number of reported TBE cases increased from 254 in 2001 to a maximum of 546 in 2006 and the number of areas at risk for TBE transmission increased from 96 in 2005 to 137 in 2012 [6]. Epidemiological analyses of the two decades from 1991 to 2000 and 2001 to 2010 in Germany show a significant (*P* < 0.001) increase in morbidity to 199.4% [2]. 410 TBE infections were reported in 2013 [7]. In Bavaria and Baden-Wurttemberg, 0.5 to 2% of unfed ticks have been shown to be TBEV-infected. However, in fed ticks removed from humans, TBEV RNA was detected in 7 to 20% in endemic areas of high risk, indicating that the infection rate in fed ticks can be up to 21.5 times higher than in unfed ticks. On this basis it has been hypothesized that a blood meal leads to an increase in virus replication [5].

Over the last few years, the TBE virus has been documented to be spreading into regions where it was not previously endemic. Not only has it been detected in more northern areas such as Denmark and Norway, it has also been found at higher altitudes, including the Krkonose Mountains in the Czech Republic and the Austrian Alps [2, 4, 8–11]. In July 2008, six human TBE virus infections occurred after consumption of unpasteurized goat's milk at an altitude of 1,500 meters a.s.l. [8]. The incidence of TBE depends on the density of infected host-seeking ticks, the level of exposure, and the vaccination rate of the population [5, 12, 13].

**Figure 1:**
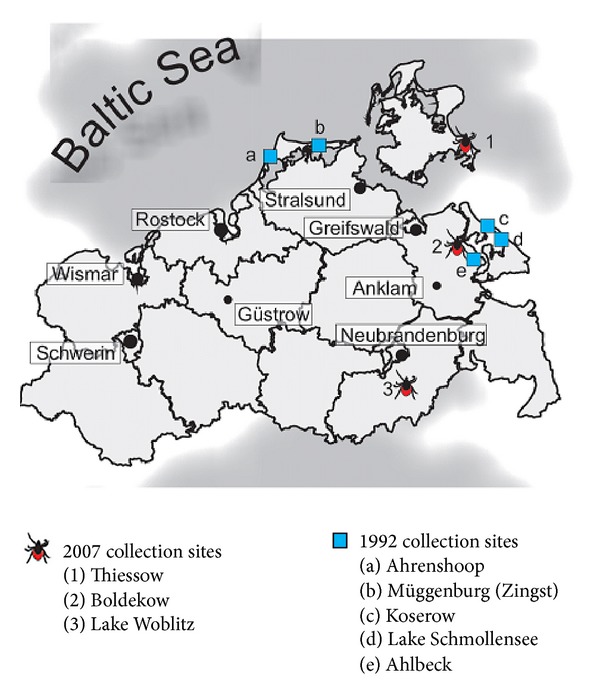
Tick-collection sites from 2007, where autochthonous cases of TBE have appeared in Mecklenburg-West Pomerania since 2004 and natural TBEV foci of 1992.

Mecklenburg-West Pomerania had never been declared as an area of risk according to the definition of the public health authority, but few autochthonous cases and TBEV-RNA detection in ticks showed a low activity of the virus in the past: from 1960 to 1985, four human cases of TBE were reported east of the town Neustrelitz in Mecklenburg-West Pomerania [6, 14, 15], and natural foci of TBEV were detected in tick pools in North East Germany using RT-PCR in 1992 [14, 16] (Figure 1). Between 1992 and 2003, a total of 16,089 ticks tested negative for TBEV in Mecklenburg-West Pomerania and it was thought that TBEV had disappeared from this area (Health Department of the State of Mecklenburg-West Pomerania, unpublished data) [17].

Then, in 2004, the first autochthonous case of TBE in Mecklenburg-West Pomerania for 19 years was reported from Lake Woblitz (near Neustrelitz), to be followed by one case in the village of Boldekow near Anklam and one in Thiessow on the island of Ruegen [18, 19] (Figure 1). This led us to search for natural foci of TBEV in the regions where these cases appeared.

The aim of this study was to evaluate the prevalence of TBE virus in fed and unfed nymphs of* Ixodes ricinus* in Mecklenburg-West Pomerania.

## 2. Animals, Material, and Methods

Between February and May 2007, 300* Ixodes ricinus* ticks were collected by flagging in the regions of Lake Woblitz (Neustrelitz), Boldekow near Anklam, and Thiessow on the island of Ruegen (Figure 1). 50 unfed nymphs from each region were processed immediately as described below or frozen separately at −80°C. Another 50 nymphs from each region were put to feed on mice.

Tick-feeding chambers were prepared as follows: 10 ml syringes (Becton-Dickinson, Franklin Lakes, NJ, USA) were cut to a length of 10 mm and fixed with gauze to the proximal back of a 12-week-old immunocompetent NMRI (Naval Medical Research Institute) mouse from conventional (open-caged) housing, with health status control. Supplier of the mice: Harlan Laboratories, Inc. (Rossdorf, Germany). Ten* Ixodes ricinus* nymphs were placed together in one feeding chamber for five days. The engorged ticks were then removed with pointed tweezers and used for RNA isolation as described below or frozen separately at −80°C. Only 100 of 150 fed ticks were engorged and alive when we removed the feeding chambers and were processed as described below.

Each tick (fed and unfed) was processed separately to avoid possible RNA dilution by pooling. Each tick was homogenized using a sterile micropistill (Eppendorf, Hamburg, Germany) and then mixed in 200 *µ*l sterile 0.9% NaCl solution in a 1.5 ml tube. RNA and DNA isolation was performed using the DNeasy blood and tissue kit (Qiagen, Hilden, Germany) according to the manufacturer's instructions without adding RNase to the procedure. This method was chosen in order to copurify DNA from ticks for further studies. After isolation, a nested RT-PCR was performed as described [20], using the primers Pp1 (5′-GCG TTT GCT TCG GAC AGC ATT AGC-3′) and Pm1 (5′-GCG TCT TCG TTG CGG TCT CTT TCG-3′) for the first PCR step and Pp2 (5′-TCG GAC AGC ATT AGC AGC GGT TGG-3′) and Pm2 (5′-TGC GGT CTC TTT CGA CAC TCG TCG-3′) for the second PCR step. 5 *µ*l of each PCR product was analyzed by electrophoresis on 1% tris acetate EDTA (TAE) gel. The positive PCR products were then excised from the gel and transferred separately to 1.5 ml tubes, where they were purified using the gel extraction kit (Qiagen, Hilden, Germany). The DNA concentration was measured using GeneQuant II (Pharmacia Biotech, Freiburg, Germany) and the PCR products were sequenced (MWG Biotech, Ebersberg, Germany).

A DNA sequence analysis was performed using BLAST version 2.2.18 (National Center of Biotechnology and Information; Bethesda, MD, USA) and MEGA 4.0 (Center for Evolutionary and Functional Genomics, NCBI, Tempe, AZ, USA) to confirm TBEV subtypes and detect point mutations. As reference strains, the TBEV sequences Neudoerfl (U27495.1), Salem (FJ572210.1), Hypr (U39292.1), and Toro-2003 (DQ401140.2) were used.

A phylogenetic analysis was carried out using the program CLC main workbench version 5.0 (CLC bio, Aarhus, Denmark). A phylogenetic tree was created using the neighbour joining method (1000 replicates).

Statistical analysis was performed with SPSS 11.0 (SPSS Inc., Chicago, IL, USA). TBEV prevalence among fed and unfed nymphs from each region was compared using Fisher's exact test.

## 3. Results

A total of 250* Ixodes ricinus* nymphs were processed. RNA was isolated from 50 unfed ticks from each region and from those ticks which were alive and intact after feeding (27 from Lake Woblitz, 39 from Thiessow, and 34 from Boldekow).

A total of six ticks (2.4%) were tested positive for TBEV: three of 50 unfed (6%) and one of 27 fed nymphs (3.7%) from Lake Woblitz, along with one of 50 unfed (2%) and one of 39 fed nymphs (2.6%) from Thiessow. Neither fed nor unfed nymphs from Boldekow were TBEV-RNA positive. The difference between the infection rates in fed and unfed ticks was not significant (*P* > 0.05).

**Figure 2:**
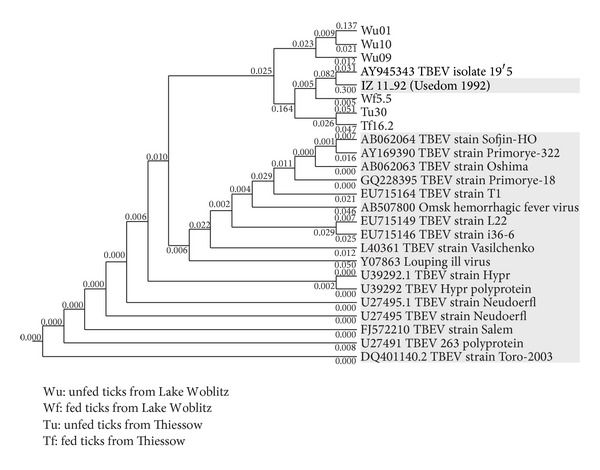
Phylogenetic tree (neighbour joining method) for 25 isolates of* Flavivirus*. Branch lengths reflect relative genetic distances between isolates. Figures at branching points represent bootstrap values. Isolate names in grey boxes designate type references for particular genetic assemblages.

The RNA sequences detected were broadly homologous to described TBEV strains, as the phylogenetic analysis shows (Figure 2). The sequence Wu 01 (tick from Lake Woblitz, unfed) was shown to be closely related to the tick-borne encephalitis virus isolates Neudoerfl (U27495.1), Salem (FJ572210.1), Hypr (U39292.1), and Toro-2003 (DQ401140.2) (92% homology in every case).

Wu 09 (Lake Woblitz, unfed) showed 100% homology to the TBEV isolates Neudoerfl, Salem, Hypr, and Toro-2003.

Wu 10 (Lake Woblitz, unfed) showed 98% homology to the TBEV isolates Neudoerfl, Salem, Hypr, and Toro-2003.

Wf 5.5 (Lake Woblitz, fed) showed 100% homology to the TBEV isolates Neudoerfl, Salem, Hypr, and Toro-2003.

Tu 30 (Thiessow, unfed) was 100% homologous to TBEV Hypr and TBEV Toro-2003 and 99% homologous to TBEV Neudoerfl and TBEV Salem.

Tf 16.2 (Thiessow, fed) showed 100% homology to the tick-borne encephalitis virus isolates Neudoerfl, Salem, Hypr, and Toro-2003.

The phylogenetic analysis revealed a close relationship of all six positive sequences and a TBEV sequence (IZ11/92) from Mecklenburg-East Pomerania obtained in 1992 (Süss 1997). The results of the phylogenetic analysis of the sequence data are shown in Figure 2.

## 4. Discussion

Six of 250 ticks (2.4%) were TBE virus-positive in the nested RT-PCR. This is the first proof of natural TBE foci in Mecklenburg-West Pomerania since 1992. In 1992, Süss et al. analyzed 18,760 unengorged ticks subdivided into 260 pools using n-RT-PCR and southern blot hybridization. Two tick pools from the Darss peninsula (near the villages Ahrenshoop and Müggenburg) and three tick pools from the island of Usedom (the villages Ahlbeck, Schmollensee, and Koserow) were found to be TBEV-positive, and one sequence was published (IZ-11/92) [14, 16, 21] (Figure 1). In the aftermath of this study over 16,000 ticks were collected between 1993 and 2003 but none was found to be TBEV-positive, leading to the assumption that natural foci were extinct or only present at an extremely low level of activity (Health Department of the State of Mecklenburg-West Pomerania, unpublished data) [17].

In 2004, a clinically proven case of TBE infection was reported from a campsite near Groß Quassow on Lake Woblitz [15], and in 2007 we collected and analyzed ticks from this region. Four of 77 (5.2%) ticks were TBEV-positive with nested RT-PCR. In Thiessow in the south-east of the island of Ruegen, where another autochthonous case occurred in 2005, two out of 89 (2.2%) ticks were TBEV-positive with nested RT-PCR. In Boldekow near Anklam where the third autochthonous case was reported, all 84 ticks we investigated tested negative for TBEV.

The TBEV sequences obtained displayed a high level of homology with the European prototype strain Neudoerfl and distinct western TBEV subtype isolates (Figure 2). The close relationship between the sequences we found and the single published sequence from Usedom from the year 1992 (IZ-11/1992) may indicate that the TBEV-foci in Mecklenburg-West Pomerania persisted over the years at low levels of activity. Data for the remaining sequences from 1992 from Usedom and the Darss peninsula are, unfortunately, not available (Figures 1 and 2) [14, 16].

This first evidence of TBEV in ticks for 15 years might be explained by warmer winter temperatures leading to an increase in tick activity [22]. The winter of 2006/2007 was the warmest in Germany since annual temperature statistics began to be recorded in 1901. An average temperature of 4.6°C was measured in Mecklenburg-West Pomerania, a deviation of +4.4°C from the previous longstanding average temperature [23].

A possible explanation for the relatively high prevalence of TBEV-RNA in nymphs in our study compared to the unsuccessful detection of TBEV-RNA in the previous years in Mecklenburg-West Pomerania may lie in the fact that we processed the ticks separately without pooling them. The widespread practice of detecting TBEV-RNA by PCR from pools of 3 to 10 adult ticks or pools of up to 200 nymphs may dilute the total RNA concentration of the specimen below the detection limit. In geographic regions with relatively low TBEV prevalence in particular, this may lead to a distortion of results and a bias towards TBEV-negativity.

The TBEV detection rate has been reported to be up to 21.5 times higher in ticks fed on humans than in unfed ticks [5]. On the basis of these data, we expected the prevalence of TBEV to be higher in our fed ticks due to the hypothetical increase in virus replication during the blood meal. However, we were unable to determine a significant difference in TBEV infection rates in fed versus unfed nymphs for either Lake Woblitz (1 versus 3) or Thiessow (1 versus 1) in our study.

PCR-inhibitors from blood may have biased the results, though according to the DNA and RNA isolation protocol, inhibitors should have been sufficiently eliminated. Cofeeding (feeding closely together on the same animal) has been shown to support virus transmission from infected adults to uninfected nymphs [24, 25] but we did not observe this in our study (which anyway involved the feeding of nymphs only).

Our results imply that natural TBEV foci can latently persist at low levels of activity for years. This is exemplified by the area around Lake Woblitz, near Neustrelitz, which was a focus of TBE between 1960 and 1985, during which four clinical cases were reported, after which the next case did not occur until 2004 [2, 14, 15]. The close relationship between the sequences reported in this study and a TBEV strain found on the island of Usedom in 1992 supports this hypothesis. Another possible scenario is that TBEV may have reemerged in extinct natural foci through the agency of migrating birds. This hypothesis could also apply to the emergence of TBEV in Thiessow on the island of Ruegen. Together with other coastal areas of Mecklenburg-West Pomerania, Ruegen is a rest stop for more than 27 species of water bird and over five million migratory birds annually along the Atlantic flyway [26]. The theory that migrating birds act as hosts and transport media for ticks could also explain TBE in another setting in South West Germany, where at a monkey park in 2006 a closely related TBEV strain was isolated from the brain tissue of an infected, naturally exposed monkey (*Macaca sylvanus*) [27, 28].

Further studies using unpooled ticks from Mecklenburg-West Pomerania are needed if we are to obtain complete prevalence data for this region and detect possible new risk areas for TBE infection early. Examining TBEV reservoirs, such as mice, goats, and sheep, which can function as sentinels, and returning to explore the natural foci described in 1992 may generate results that would serve as a useful addition to the present data [29].
